# Local Immune Responses of the Chinese Water Buffalo, *Bubalus bubalis*, against *Schistosoma japonicum* Larvae: Crucial Insights for Vaccine Design

**DOI:** 10.1371/journal.pntd.0002460

**Published:** 2013-09-26

**Authors:** Hamish E. G. McWilliam, David Piedrafita, Yuesheng Li, Mao Zheng, Yongkang He, Xinling Yu, Donald P. McManus, Els N. T. Meeusen

**Affiliations:** 1 Biotechnology Research Laboratories, School of Biomedical Sciences, Monash University, Melbourne, Victoria, Australia; 2 Australian Research Council Centre of Excellence in Structural and Functional Microbial Genomics, Monash University, Melbourne, Victoria, Australia; 3 Hunan Institute of Parasitic Diseases, Yueyang, Hunan, China; 4 Molecular Parasitology Laboratory, Queensland Institute of Medical Research, Brisbane, Queensland, Australia; University of York, United Kingdom

## Abstract

Asian schistosomiasis is a zoonotic parasitic disease infecting up to a million people and threatening tens of millions more. Control of this disease is hindered by the animal reservoirs of the parasite, in particular the water buffalo (*Bubalus bubalis*), which is responsible for significant levels of human transmission. A transmission-blocking vaccine administered to buffaloes is a realistic option which would aid in the control of schistosomiasis. This will however require a better understanding of the immunobiology of schistosomiasis in naturally exposed buffaloes, particularly the immune response to migrating schistosome larvae, which are the likely targets of an anti-schistosome vaccine. To address this need we investigated the immune response at the major sites of larval migration, the skin and the lungs, in previously exposed and re-challenged water buffaloes. In the skin, a strong allergic-type inflammatory response occurred, characterised by leukocyte and eosinophil infiltration including the formation of granulocytic abscesses. Additionally at the local skin site, interleukin-5 transcript levels were elevated, while interleukin-10 levels decreased. In the skin-draining lymph node (LN) a predominant type-2 profile was seen in stimulated cells, while in contrast a type-1 profile was detected in the lung draining LN, and these responses occurred consecutively, reflecting the timing of parasite migration. The intense type-2 immune response at the site of cercarial penetration is significantly different to that seen in naive and permissive animal models such as mice, and suggests a possible mechanism for immunity. Preliminary data also suggest a reduced and delayed immune response occurred in buffaloes given high cercarial challenge doses compared with moderate infections, particularly in the skin. This study offers a deeper understanding into the immunobiology of schistosomiasis in a natural host, which may aid in the future design of more effective vaccines.

## Author Summary

Schistosomiasis is caused by a parasitic blood fluke, and in parts of Asia it infects both humans and livestock such as water buffaloes. This makes controlling the disease more difficult, because both humans and livestock must be treated regularly. A vaccine given to buffaloes is likely to reduce human infection rates and improve buffalo health by providing long-lasting protection from re-infection; at present no vaccines are available. Older buffaloes are known to have some immunity to schistosomiasis which is acquired over time; however how this occurs is not understood. In this study we investigated the immune response of buffalo against the schistosome larvae, which are vulnerable to immune attack, and hence are the ideal stage to target for vaccination. We found that the buffalo produces a profound allergic type-2 response as larvae penetrate the skin, with significant cellular infiltrates and abscesses. When the larvae move next to the lungs, a uniquely type-1 response was induced. This skin response is much greater than more susceptible animals such as mice, and may be a mechanism for larval killing in the buffalo. This study offers insight into the immunobiology of an important host for schistosomiasis and may help in designing better vaccines.

## Introduction

Asian schistosomiasis, caused by *Schistosoma japonicum*, is a parasitic disease endemic in the marsh and lake regions of China, particularly along the Yangtze River basin and mountainous regions [1], [2], and parts of the Philippines and Indonesia [3]. Up to 1 million people are infected with 50 million at risk of infection [1], [4]. Schistosomiasis causes significant morbidity in chronically infected individuals, perpetuating poverty in endemic communities [5], and is considered to be one of China's major public health priorities [6].

Control of Asian schistosomiasis relies largely on chemotherapeutic treatment with praziquantel (PZQ) however this is complicated by the fact that it is a zoonotic disease and infects a wide variety of mammalian hosts [7], [8]. In particular, the domestic water buffalo, *Bubalus bubalis*, is considered the most significant animal reservoir of schistosomiasis in China [1], [9], [10] where it has been shown to account for up to 75% of human transmission [11]. This results in the need for routine PZQ treatment of livestock in addition to humans for parasite control [1]. However as a complementary strategy, a vaccine could contribute significantly to the elimination of schistosomiasis [1], [12], [13], [14], [15], [16], [17], [18]. An effective vaccine for buffalo alone would significantly reduce human morbidity as well as improve buffalo health [19], and for this reason an effort has been made to develop a transmission-blocking veterinary vaccine [17].

Much of our understanding of the immunobiology of schistosomiasis comes from murine models [20] but there is a distinct lack of understanding of the immunological mechanisms during schistosomiasis in natural large animal hosts, including water buffaloes. Moreover the vast majority of previous studies were performed in naive mice, and there is limited information on the response from endemically-exposed hosts, which are the intended recipients of a vaccine. There are several reports of age-related acquired resistance in buffaloes that show that older buffaloes have reduced intensity and prevalence of infection [2], [9], [21], [22]. Also, buffaloes are known to have a natural resistance to *S. japonicum* infection compared with other hosts, such as cattle, *Bos taurus* [8], [23]. While this acquired and natural immunity has been documented, it has not been studied in depth and the mechanism is unknown. Since the migrating schistosomula are recognised as a susceptible target in humans and other animals [14], [24], [25], [26], it is likely that this stage may be also targeted by immune effector mechanisms in buffaloes.

In order to elucidate the immunobiology of schistosomiasis in an endemic host, we investigated the local immune response directed at the migrating schistosome larvae in water buffaloes from an endemic area in China. We characterised the type of immune response at each local site of larval migration, the skin and the lungs, in comparison with the liver where the majority of eggs are trapped. The results of this study may shed light on a mechanism of immunity in a natural host of *S. japonicum* and inform the design of more effective vaccines.

## Methods

### Ethics statement

All experimental animal procedures were performed with written approval from the Ethical Review Board of the Hunan Institute of Parasitic Diseases (approval no. 110818), and from the Monash University Animal Ethics Committee (no. 2011-124-FW). The handling and care of the animals were performed by trained staff adhering to good animal practice guidelines according to the Animal Ethics Procedures and Guidelines of the People's Republic of China.

### Animals and experimental plan

Eleven mixed-sex (8 male and 3 female) water buffaloes (*B. bubalis*) were selected from a schistosomiasis-endemic area in Hunan Province, aged between 1–1.5 years (Table 1). Animals previously exposed to *S. japonicum* were selected based on positive faecal egg counts, which required testing over 50 buffaloes from the region. Animals were then moved to *S. japonicum*-free pasture in a non-endemic region of Hunan Province to rest for 7 weeks, during which time they were treated with praziquantel (PZQ) twice to cure the existing schistosomiasis. Because PZQ is only effective on adult worms older than 4 weeks, the second dose was to cure any parasites acquired immediately prior to being moved to the parasite-free pasture. Subsequent to the second PZQ treatment the miracidial hatch test was performed [27] and was negative for all buffaloes, indicating they were no longer excreting viable eggs.

**Table 1:** Experimental trial and individual buffalo data.

Group	Buffalo ID#	Infection dose (cercarial number)	Sex	Age (years)	Initial faecal egg count∧	Weight (kg)	DPI sacrificed
NI	1	-	M	1.3	2.0	165.8	-
	2	-	F	1.5	1.0	193.5	-
	3	-	M	1.2	1.0	137.1	-
5–6 DPI	1	Moderate (200)	M	1.0	0.2	136.3	6
	2	Moderate (400)	M	1.5	1.0	223.7	6
	3	Moderate (600)	F	1.4	1.0	181.0	5
	4	High (2400)*	M	1.1	2.0	134.5	5
11–12 DPI	1	Moderate (200)	M	1.5	0.5	202.5	11
	2	Moderate (400)	F	1.4	2.0	170.1	11
	3	Moderate (600)	M	1.5	0.5	229.8	12
	4	High (2400)*	M	1.5	0.3	200.9	12

NI = not infected; DPI = days post infection.

∧ Faecal egg count (eggs per gram of faeces) measured at the time the buffaloes were acquired, prior to praziquantel treatment;

* High-dose infection animals were considered separately from their respective group in the analyses.

Eight buffaloes were chosen randomly for experimental infections, and these were divided into two groups for sacrifice at two time points; either at 5 and 6 days post-infection (5–6 DPI) to study the skin-penetrating cercariae, or 11–12 DPI to focus on the lung-migrating larvae. These particular time points were chosen to study the matured adaptive response in the local draining lymph node (LN), since the peak of an adaptive immune response occurs around 4–5 days in draining LN following antigenic challenge [28], [29]. The response in the skin-LN was likely to be maximal at 5–6 DPI while 11–12 DPI was chosen to study the response against the lung-stage of infection, as the peak of larvae migration through the buffalo lung is around 7 DPI (unpublished observations). Three buffaloes were left un-infected (group NI) and sacrificed at different days throughout the trial (−1, 5 and 11 DPI).

Infection with *S. japonicum* cercariae was administered percutaneously on the shaved inner thigh, by transferring cercariae with a sterile culture loop onto a glass cover slip, and then resting this on the moistened skin for at least 30 minutes. The *S. japonicum* cercariae were obtained from *Oncomelania hupensis* snails collected from the marshlands of the Dongting Lake Region, Hunan Province.

Three buffaloes in each challenge group were given a moderate infection of 200–600 cercariae, depending on availability (Table 1). Matched pairs were given the same cercarial number, and numbered according to this dose. One buffalo at each time point (#4) received a high dose of 2400 cercariae to be used as a source of highly-stimulated samples for a separate study.

### Sample collection

At post-mortem, lymph nodes (LN) draining the skin of the inner thigh (inguinal LN), lungs (mediastinal LN) and liver (portal LN) were collected and sliced into pieces in a sterile petri dish containing approximately 5 ml of media (RPMI 1640 Glutamax, supplemented with penicillin (100 U/ml) and streptomycin (100 µg/ml) (Life Technologies)). These were stored on ice until processed. A sample of skin (∼1 cm^2^) was removed from the site of parasite infection, as was a similar piece of lung tissue. Half of each was placed into RNAlater (Ambion), and the other half fixed in 10% formal saline for up to 2 weeks, followed by storage in 70% ethanol until they were processed into paraffin for histological analysis.

### Lymph-node cell stimulations

Lymph node slices were gently teased apart in cold media under sterile conditions. Cells were washed twice in cold media, each time collected by centrifugation at 400× *g* for 10 min at 4°C. Lymphocytes were counted using a haemocytometer and trypan blue to exclude dead cells, and were resuspended to 1×10^7^ cells/ml in media supplemented with AlbuMAX II (2 mg/ml; Life Technologies). Lymphocytes were cultured for 48 hours at 37°C with 5% CO_2_ and stimulated by adding phorbol myristate acetate (PMA; 10 ng/ml) and ionomycin (1 µg/ml). After culturing, the cell supernatants were collected and stored at −20°C until required.

### Skin histology

Fixed skin samples were paraffin-embedded and 4 µm sections were cut and stained with haematoxylin and eosin (H&E) for leukocyte counting, toluidine blue for mast cell differentiation, or stained overnight with Llewellyn's Sirius red for eosinophil identification [30]. Cells were counted in 5–7 different fields-of-view at 400× magnification to determine the numbers of these cell types in the skin. To count the number of leukocytes in the epidermis, 5 sections of 0.26 mm in length were enumerated. To determine the thickness of the epidermis and stratum corneum layers, ImageJ [31] was used to calculate the volume of each layer relative to the length of the skin section (determined by measuring the stratum granulosum). For each sample, three sections of skin (approximately 0.8 mm) were measured. Skin eosinophilic abscesses were avoided for the cell counts since individual cells could not be distinguished.

### Measuring cytokine transcript levels in skin by quantitative real-time polymerase chain reaction (qPCR)

Buffalo skin samples were disrupted using a T10 basic Ultra-Turrax homogeniser (IKA) in the presence of Qiazol (Qiagen), and the RNA was subsequently extracted according to Qiagen's protocol. After resuspending the RNA pellet in water, it was purified using the Total RNA Extraction Miniprep System (Viogene) and the concentration determined on a NanoDrop spectrophotometer (Thermo Scientific). Total RNA (90 ng) was converted to cDNA using the QuantiTect Reverse Transcription Kit (Qiagen) which includes a genomic DNA removal step. To perform the qPCR, primers were used based on *B. bubalis* sequences [32], except for galectin-14 which was based on the ovine sequence since the *B. bubalis* sequence was not available. The sequences of the primers are shown in Table 2. The qPCR reactions included SYBR Master Mix (Applied Biosystems) and the above primers at 0.5 µM and 5 µl of 1∶20 diluted cDNA, and were run in triplicate on an Eppendorf Realplex4 Mastercycler for 40 cycles, using the annealing temperatures (Tm) shown in Table 2. Melt curve analysis was done to ensure a single product was amplified. The relative copy number of SYBR green for each gene was calculated from a standard curve of serial dilutions of cDNA, and then the relative expression of the target gene was determined relative to the reference gene copy number.

**Table 2:** Primer sequences for qPCR.

Target		Primer sequence (5′-3′)	Tm (°C)	Product length (bp)
β-actin	F	CGCACCACCGGCATCGTGAT	60	227
	R	TCCAGGGCCACGTAGCAGAG		
IFN-γ	F	GTCTCCTTCTACTTCAAACT	55	255
	R	ATTCTGACTTCTCTTCCGCT		
IL-10	F	CTGCTGGATGACTTTAAGGG	60	187
	R	AGGGCAGAAAGCGATGACAG		
IL-4	F	CAAAGAACACAACTGAGAAG	53	121
	R	AAGCTGTTGAGATTCCTGTC		
IL-5	F	TGGCAGAGACCTTGACACTGCT	60	164
	R	CACAGCATCCCCTTGTGCAGTT		
Galectin-14	F	CACCCAGCCTCCCTACATAA	58	144
	R	ATGCCCATCTGAAAGTCCAC		

### Measuring cytokine protein levels in cell culture supernatants

The levels of IFN-γ, IL-4 and IL-10 were quantified in cell culture supernatants by sandwich ELISA. To determine IFN-γ levels, the Bovine IFN-γ ELISA kit (AbD Serotec) was used according to the manufacturer's instructions. For IL-4 and IL-10, monoclonal antibody pairs were used (AbD Serotec). Plates were coated with mouse anti-bovine IL-4 or IL-10 monoclonal antibody (2.5 µg/ml) overnight at 4°C. All subsequent steps were done at room temperature, and washing consisted of 5 changes of PBS with 0.05% Tween-20 (PBST). After blocking with 0.1% bovine serum albumin in PBS for 1 hour, samples were incubated for 1.5 hours (the supernatants or recombinant bovine IL-4/IL-10 for standard curves). The supernatant samples were diluted 1∶20 for IL-4 and IFN-γ and 1∶50 for IL-10 measurement. After washing, biotinylated anti-bovine IL-4 or IL-10 was added at 1∶1600 or 1∶1000, respectively, and incubated for 1 hour and subsequently washed. Finally, horseradish peroxidise-conjugated streptavidin (Dako) was added at 1∶1000 for 1 hour, and plates were developed using TMB Substrate (Life Technologies) and optical density measured using a spectrophotometer. Supernatant OD readings were compared to the linear part of the standard curve to determine the cytokine amount.

### Data interpretation and statistical analysis

This study was limited in the sample sizes that could be included for each group, due to the high cost, the large scale and the logistics involved in using water buffaloes. Therefore many of the differences are described as trends, and statistical results are only reported when significance was achieved at p<0.05. When comparing three groups, a one-way analysis of variance test followed by Tukey's post-hoc test was performed, and when comparing two groups the student's t-test was employed. To measure correlations between two parameters, the Spearman's rank correlation was performed and the rank coefficient (*r_s_*) was determined. Considering that the two high-dose buffaloes (#4) received up to 12 fold more cercariae than the moderate infections, their data are not included in the means or statistical analyses but are represented separately in the figures.

## Results

### Gross observations at necropsy

**Figure 1:**
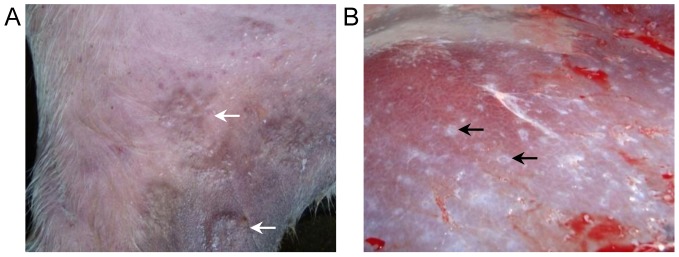
Gross skin and liver observations from infected buffaloes. Buffaloes were infected on the inner thigh with *S. japonicum* cercariae and 5 days post-infection this resulted in raised inflammatory reactions (particularly for buffalo #4 which received the highest cercarial challenge) (A). At necropsy livers showed evidence of egg-induced pathology (B). White arrows indicate the raised skin reaction, while black arrows highlight the white nodules of egg pathology.

Buffaloes were infected via the cover-slip method on the inner thigh and at 5 DPI the skin site where cercariae were applied had an obvious raised inflammatory reaction (Fig. 1A). By 11–12 DPI the skin reaction had reduced and was no longer raised. No difference was seen in the lungs between infected and uninfected buffalo, at either time point (data not shown). All buffaloes were selected based on positive *S. japonicum* faecal egg counts (see Table 1), and this previous infection history was reflected in the gross morphology of the livers where white nodules were visible (Fig. 1B). All livers had a similar appearance due to these prior infections, which is typical of a patent bovine schistosome infection [33].

### Cellular and cytokine responses at the skin site of larval penetration

**Figure 2:**
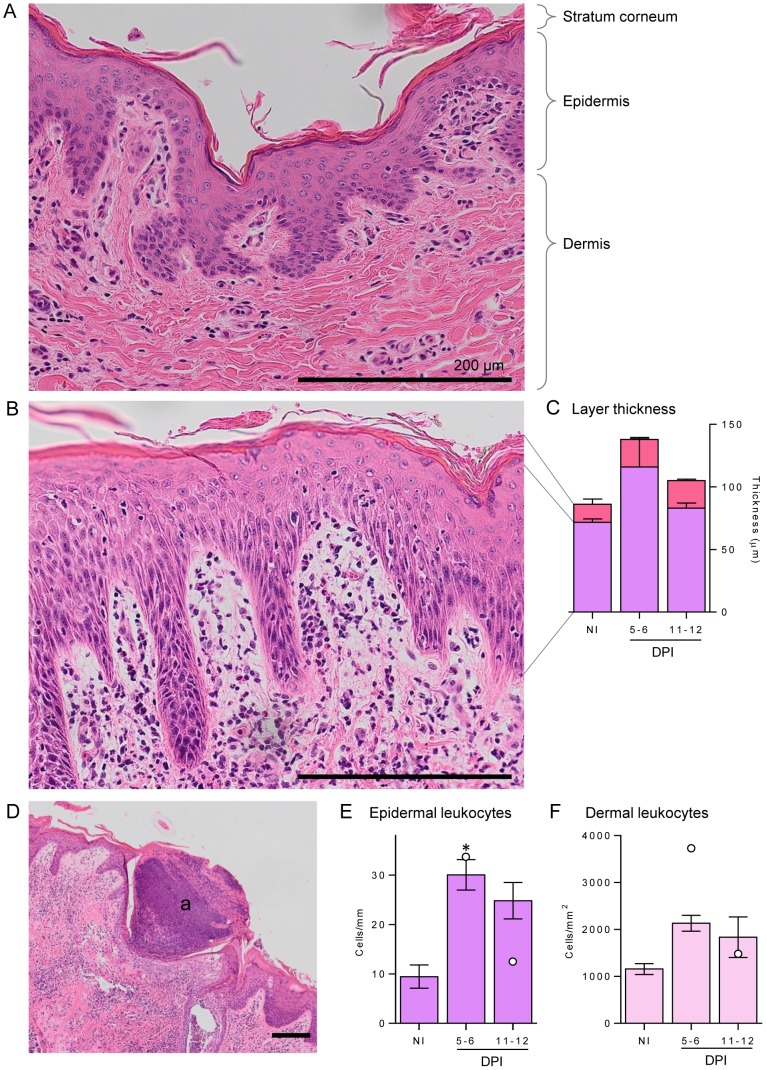
Leukocyte infiltration and thickness of buffalo skin following infection. A buffalo skin section from non-infected buffaloes (A) are compared to those following moderate infection with *S. japonicum* cercariae (B and D), which resulted in a large influx of leukocytes in dermal and epidermal layers (B) and granulocytic abscesses (a) formation (D). Stratum corneum, epidermis and dermis are indicated for reference. Staining was performed with haematoxylin and eosin, and all scale bars represent 200 µm. Leukocytes were enumerated in the epidermis (E) and dermis (F) in non-infected (NI) and 5–6 or 11–12 days post-infection (DPI) buffaloes. For each buffalo 5 fields of view of the dermis at 400× magnification were enumerated and averaged, while for the epidermis 5 lengths of 0.26 mm were enumerated and averaged. The thicknesses of the epidermis (purple) and stratum corneum (pink) (C) were measured by determining volume of each layer relative to the length of the skin section, and 3 sections of 0.8 mm were averaged for each individual. Bars represent the mean of the moderate infections with standard error; the two high infections are shown in E and F as white dots. Significance above NI levels is shown by the asterisk (p = 0.015).

Compared with uninfected controls, the skin of infected buffaloes at both time points showed signs of strong inflammation (Fig. 2), characterised by large numbers of infiltrating leukocytes in infected (Fig. 2B) compared with control skin (Fig. 2A). Thickening of the two outer layers, the epidermis and stratum corneum, was observed at 5–6 DPI (Fig. 2C); although not statistically significant, the thickness of all infected skin samples were above NI values. The epidermis was thickest at 5–6 DPI and then reduced to near NI level at 11–12 DPI, whereas the thickened stratum corneum increased slightly further at 11–12 DPI. Large, eosinophilic abscesses were seen in all skin sections at 5–6 DPI (Fig. 2D), in most cases severely disrupting the architecture as they spanned multiple skin layers; these had mostly cleared up at the later time point with only one of the four buffaloes from 11–12 DPI group (#1) showing abscesses. These abscesses consisted of dense nuclei, likely to be infiltrating leukocytes, with eosinophilic granules staining throughout (not shown). Leukocytes were enumerated in the epidermal (Fig. 2E) and dermal layers (Fig. 2F) and showed a similar pattern: a large increase in leukocytes initially at 5–6 DPI and a slight reduction at 11–12 DPI. This was significantly higher only in the epidermal layer.

**Figure 3:**
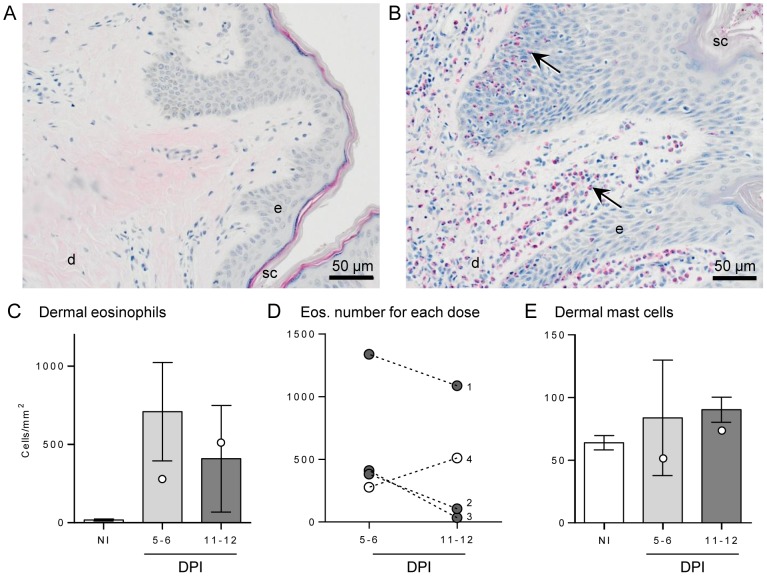
Eosinophil and mast cell numbers of buffalo skin following infection. Skin sections from a non-infected buffalo (A) and following moderate *S. japonicum* infection (B) stained with Llewellyn's sirius red stain highlights numerous eosinophils (black arrows) infiltrating the skin layers post-infection. Stratum corneum (sc), epidermis (e) and dermis (d) are indicated for reference. Eosinophils were enumerated in non-infected (NI) and 5–6 or 11–12 days post-infection (DPI) buffaloes (C). Bars represent the mean of the moderate infections with standard error; the two high infections are shown as white dots. The change in eosinophil number over time for each cercarial dose is shown in (D), where buffaloes #4 (white dots) had a high dose (2400) and #1-3 (grey dots) had a moderate dose (200–600), respectively. Numbers of mast cells in the dermis (E) were counted after toluidine blue stain. For each buffalo 7 fields of view at 400× magnification were enumerated and averaged.

A large number of infiltrating eosinophils were seen in infected buffalo skin compared with controls (Fig. 3A–C), and this was predominantly in the dermal layer. Some eosinophils were seen invading discrete sections of infected epidermis (Fig. 3B); however this was not consistent and seen in very few sections, hence numbers were not determined. Eosinophils were enumerated in the dermal layer (Fig. 3C), showing a dramatic increase from a negligible amount (19 cells/mm^2^) in control skin to 710 cells/mm^2^ of dermis at 5–6 DPI and then dropping slightly to 410 cells/mm^2^ at 11–12 DPI. When individual data points of eosinophil numbers in infected dermis from buffaloes given the same dose of infecting cercariae were linked, it was observed that for all buffaloes receiving a moderate challenge dose the number of eosinophils was highest at 5–6 DPI, reducing by 11–12 DPI (Fig. 3D). However, for the pair of buffaloes given the high dose (#4) the number of eosinophils was highest at the later time point.

Mast cells were observed in the dermis of all skin samples (Fig. 3E), including NI skin, with a consistent increase in mast cell numbers only at 11–12 DPI compared to uninfected skin; despite no statistical significance, all samples at this latest time point had a higher number of mast cells than NI sections, increasing from a mean of 64 to 90 cells/mm^2^.

**Figure 4:**
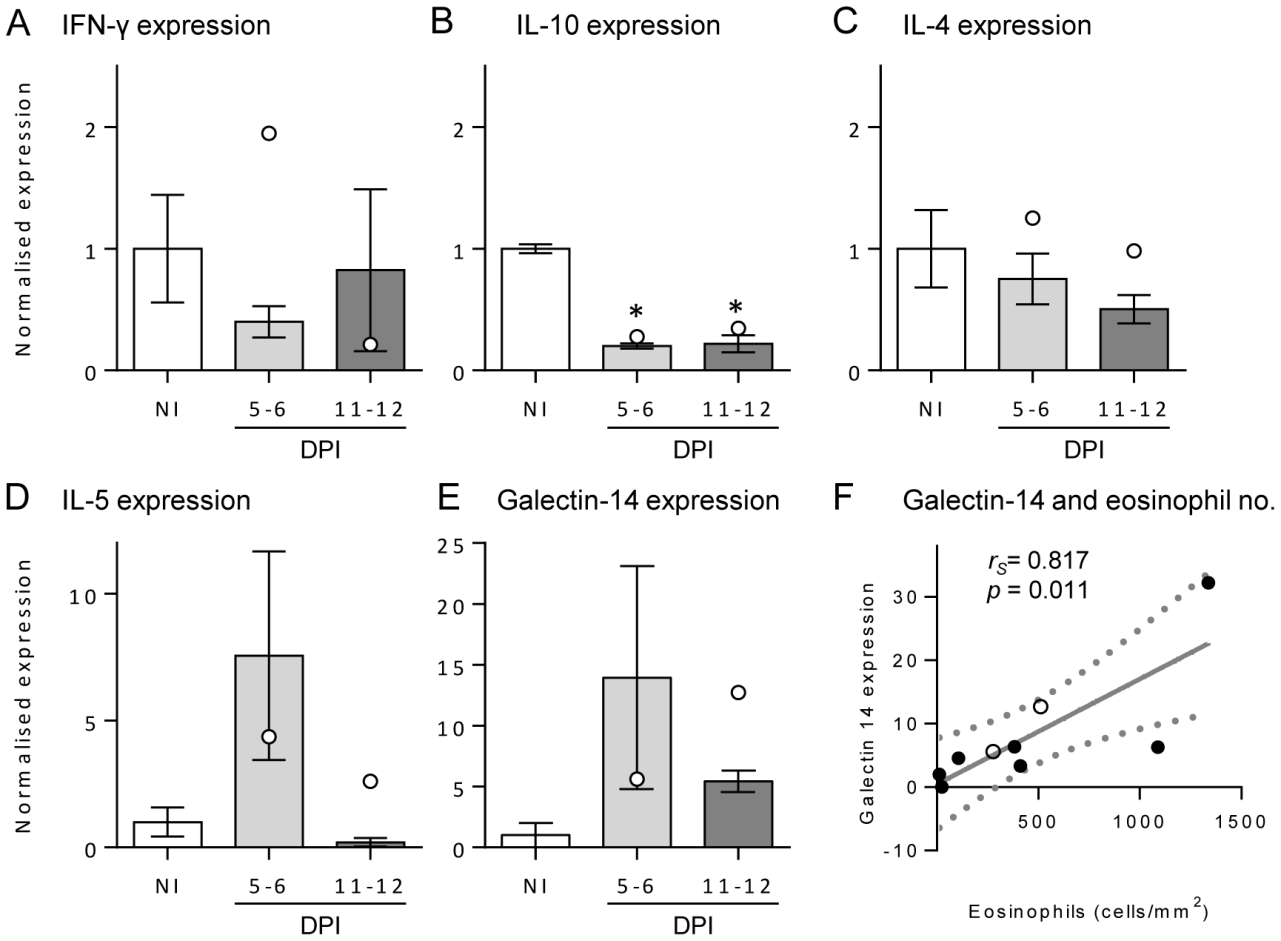
Transcript levels of IL-5, IFN-γ and galectin-14 in buffalo skin. The levels of IFN-γ (A), IL-10 (B), IL-4 (C), IL-5 (D) and galectin-14 (E) transcripts were determined in skin samples by quantitative real-time polymerase chain reaction from non-infected (NI) and 5–6 or 11–12 days post-infection (DPI) buffaloes. Each sample was normalised to the expression of β-actin, and then shown relative to the mean of the NI samples. Bars represent the mean of the moderate infections with standard error; the two high infections are shown as white dots. Significant differences from NI levels are indicated by asterisks. The level of galectin-14 transcript and eosinophil numbers in tissue sections positively and significantly correlated (F) as determined by a Spearman's rank coefficient calculation (r_s_ = 0.87, p = 0.011).

The type of immune response occurring in the skin infection site was investigated by measuring the transcript level of IFN-γ (a typical Th1 cytokine), IL-4 (a Th2 cytokine), IL-5 (a Th2 cytokine which promotes eosinophil proliferation and survival [34]), IL-10 and galectin-14 (a molecule specific to eosinophils [35]) (Fig. 4A–E). The levels of these transcripts were normalised to a reference gene (β-actin) and then calculated relative to the mean of control samples to observe changes resulting from infection. There was a slight non-significant drop in skin IFN-γ following infection, and IL-4 levels remained fairly constant in each group. At both time points post-infection, the level of IL-10 expression was reduced significantly (*p*<0.001) to approximately one-fifth of the control level. IL-5 dramatically increased to 7.5 times the level of normal skin at 5–6 DPI and then returned to the NI level at 11–12 DPI, although this was not significant (*p = *0.302). The level of galectin-14 increased to approximately 14 times the level of expression in NI skin at 5–6 DPI, reducing to 5 times the level at 11–12 DPI, also not significant (*p = *0.491). Furthermore, there was a positive and significant correlation between the number of eosinophils in the skin with the level of galectin-14 (*p* = 0.011, *r_s_* = 0.817; Fig. 4F), but not with the level of IL-5 (*p* = 0.744; data not shown). As found with the number of eosinophils in tissues, only the #4 buffaloes given the high dose had an increase in galectin-14 expression at 11–12 DPI compared to 5–6 DPI.

### Cytokine responses in draining LN

**Figure 5:**
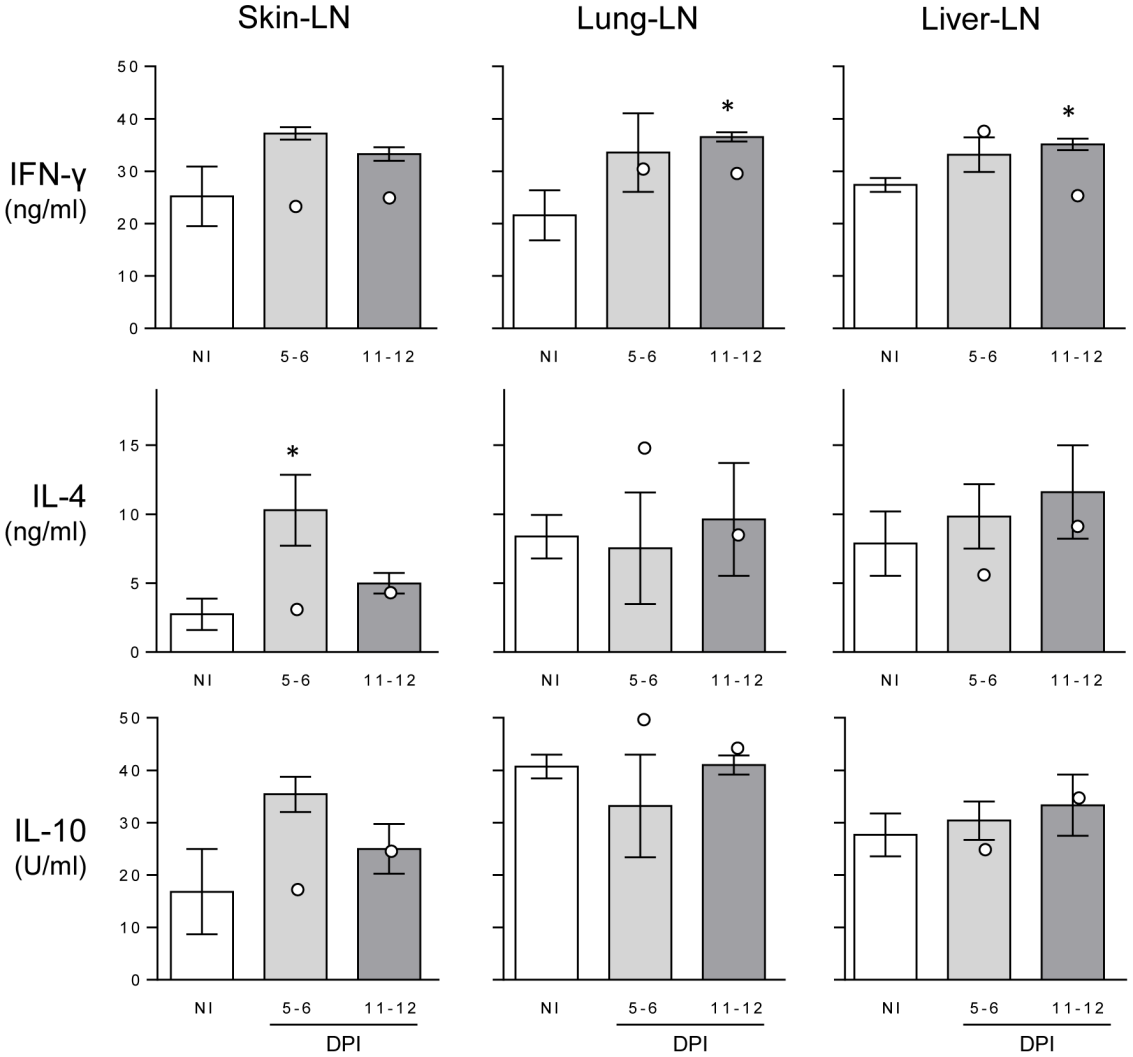
Cytokine levels in stimulated lymph node cell culture supernatants. Levels of IFN-γ, IL-4 and IL-10 were measured by ELISA from PMA/ionomycin stimulated lymphocytes taken from skin-, lung- and liver-lymph nodes (LN) from non-infected (NI) and 5–6 or 11–12 days post-infection (DPI) buffaloes. Bars represent the mean of the moderate infections with standard error; the two high infections are shown as white dots). Asterisks indicate significance above NI levels (p<0.05).

Levels of IFN-γ and IL-4, the prototypical Th1 and Th2 cytokines respectively, and IL-10, were measured in stimulated cell cultures of LN draining the skin, lung and liver, and the means for the six moderate infection dose animals are shown in Fig. 5.

For the skin-LN, there was an increase in all three cytokine levels at 5–6 DPI, which was reduced at 11–12 DPI. While only IL-4 showed a statistically significant increase at 5–6 DPI above NI levels (*p* = 0.047), all three buffaloes at 5–6 DPI had higher IFN-γ levels than NI animals and two remained higher at 11–12 DPI. Interestingly, the high-dose buffaloes had higher levels of each of these cytokines at 11–12 DPI than 5–6 DPI in the skin-LN cultures (Fig. 5), similar to the eosinophil and galectin-14 levels in the skin.

For the lung-LN response, only the IFN-γ levels showed a significant increase at the latest time point of infection compared to the NI level (*p* = 0.036); there was no change in IL-4 or IL-10 over the infection period. Among the uninfected animals, the lung-LN had a higher level of IL-4 and IL-10 than skin-LN, indicating a difference in basal stimulation of the lungs draining LN compared to those of the skin. The IFN-γ levels were consistent among each LN in NI individuals. Finally, the liver-LN response showed a trend towards an increase for each cytokine at the latest time point, although this was only significantly above NI for IFN-γ (*p* = 0.011).

## Discussion

This study investigates the local immune response against migrating schistosome larvae in the water buffalo. Despite the evidence of acquired immunity in this natural host, there have been few studies on the buffalo immune response during schistosomiasis [14], and to our knowledge none exploring the anti-larval response. In the present study, the skin and lungs were examined at two time points after cercarial infection, estimated to reflect a mature immune response in the LN draining those tissues. Overall the findings indicated that these time points did correspond with the timing of larval migration, since we observed consecutive cytokine responses; the response in the skin-LN was highest at 5–6 DPI, with the lung-LN IFN-γ highest at 11–12 DPI.

The local immune response against invading cercariae in the skin was the most obvious pathological change evident during the study and was characterised by a strong type-2 inflammatory response. Examination of the skin site showed raised allergic ‘wheal’ type reactions at 5–6 DPI, which had reduced by 11–12 DPI. Histological and qPCR analysis, as well as skin-LN cell stimulations, confirmed a predominant type-2 response against the penetrating larvae at this tissue site. Large numbers of eosinophils were observed infiltrating the dermis even as late as 12 DPI, which was confirmed by transcript levels of the eosinophil-specific galectin-14. Large eosinophilic abscesses were also observed to disrupt the skin layers in these regions, similar to those described by Incani and McLaren [36] in mouse skin after a secondary infection. They found these abscesses contained dead larvae and discarded tails at 6 hours post-infection and were associated with eosinophils. Although we did not observe trapped larvae, they may have been present at earlier time points than investigated in our study or revealed by more thorough investigation of skin samples.

The stratum corneum, which is the principle defensive layer of the skin [37], showed significant thickening at 5 DPI and remained thickened even at 11–12 DPI, and this may play a protective role from further cercarial penetration. Further examination of the skin at 11–12 DPI indicated that the larvae-associated damage had begun to heal, with several parameters returning to normal levels at this time point. The increase in mast cells at this late time point could be evidence of tissue recovery, since their numbers are known to increase in skin during wound healing [38].

The most marked changes in skin cytokine transcript levels were the increase seen in IL-5 at 5–6 DPI, and the sharp and sustained reduction in IL-10 levels at both time points. In contrast, IFN-γ and IL-4 remained fairly constant. IL-5 is predominantly secreted by Th2 cells and promotes eosinophil proliferation and survival [34], consistent with the large number of eosinophils observed at this time point. By 11–12 DPI, IL-5 levels had returned to baseline indicating the type-2 response was waning, consistent with a reduction in skin-resident eosinophils. Importantly, IL-5 responses to the vaccine candidate paramyosin have been correlated with immunity for *S. japonicum* [39] and *S. mansoni* [40].

In contrast to our findings, IL-10 has been shown to increase after cercarial penetration of naive mouse and human skin [41], [42]. As a regulatory cytokine, IL-10 limits excessive inflammation by targeting leukocytes, down regulating pro-inflammatory cytokine secretion [43]. In schistosomiasis it has been considered to be a key immunomodulatory strategy of the schistosome parasite to limit the immune response during skin penetration [44]. The lack of IL-10 induction in the buffalo skin could explain the sustained skin inflammation and suggests a significant host response difference to cercarial penetration at the local site. There was an increase in IL-10 in the skin-LN however, which has been seen similarly in other hosts [44]. Additionally in the draining skin-LN there was an increase in IFN-γ and IL-4 following infection, suggesting a mixed type-1 and -2 response. A similar mixed response has been reported following primary infection of mouse skin [41]. However in the present study, the ratio of these two cytokines suggests it was predominantly oriented towards the type-2 response, and this agrees with the allergic-type reactions visible in the skin at the same time.

In contrast to the immune events described in the skin, there was no detectable gross or histological change in lung tissue post-infection (data not shown). This could be due to the fact that the relatively small schistosomula disperse throughout the lungs, which are of considerable size in water buffalo. However, the larvae did induce a measurable and significant type-1 response in the lung-draining LN at 11–12 DPI, where the IFN-γ level increased compared with non-infected levels. There was a high IL-10 level in all lung samples including uninfected animals. This could limit the extent of the inflammatory Th1 response and reduce larval killing in the lungs because a strong lung Th1 response is considered the mechanism of immune killing in the murine radiation-attenuated vaccine model [45].

The liver-LN also had more of a type-2 response than uninfected skin-LN, likely due to trapped liver eggs from previous infections which are strong type-2 inducers [20]. Similar to the lung-LN response at 11–12 DPI, the liver-LN had a late IFN-γ response, although this was less pronounced. This slight type-1 response was possibly due to the arrival of immature worms into the liver. The timing of larvae entering the liver is likely to be shortly after leaving the lungs, as occurs in the mouse model [46], since the transit time between organ vasculature is minimal [47].

One animal at each time point was infected with a significantly higher dose of cercariae in an attempt to generate highly stimulated LN samples for a separate study. Surprisingly, in almost every parameter these high-dose buffaloes consistently had the lowest response in the skin draining lymph nodes compared to the moderate infected buffaloes. In addition, the number of eosinophils and galectin-14 transcripts in the skin, and the cytokine levels in skin-LN cultures, was greater in the high-dose animal at 11–12 DPI than at 5–6 DPI, whereas the inverse was true for each of the buffaloes receiving a moderate challenge infection. While these differences were intriguing, it should be noted that it is based on a single animal in each group and could be due to individual variation, and hence should be repeated with larger numbers to draw firm conclusions. However a possible explanation for these observations is that there is greater down-regulation of the immune response in the skin and draining LN with the higher challenge dose. It is well documented that cercarial secretions have immunomodulatory properties which reduce inflammation and facilitate immune evasion [44], and the higher number of cercariae would mean more secretions present in the skin. Additionally, while cercariae are generally thought to discard their tails before skin penetration, Wang *et al.* [48] recently showed that with high-density cercariae infections more tails enter the skin. It is possible that the products derived from these tails may also suppress or delay immune responses in the high-dose buffaloes. Doses of several thousand cercariae have been used in previous buffalo studies [23], [33], [49] and in future immunological investigations lower doses may be more appropriate.

Overall, the data show that an inflammatory type-2 response was generated following cercarial invasion of water buffalo skin, with a mixed but predominantly type-2 response in the skin draining LN. Then as the larvae migrated to the lungs, a moderate type-1 response was seen in the lung-LN. To our knowledge, this is the first time the immune response to migrating schistosome larvae has been investigated in an endemically-exposed host, shedding light on the immune mechanisms induced during a natural infection. The current paradigm of schistosomiasis holds that the migrating larvae induce a primarily Th1 response, which then becomes a strong Th2 response several weeks later after the worms mature and eggs are deposited in tissues [20]. This has largely been elucidated from primary infections in naive rodent models, where the eggs provide a strong Th2-inducing stimulus [50]. The radiation-attenuated vaccine has also been studied extensively in mice, where immunity is mostly attributed to a protective Th1 response primed in the skin, which is then effected in the lungs [24], [45], [51].

In contrast to the Th1-protective mechanism in the mouse, protection in humans from schistosomiasis-endemic communities is linked with type-2 responses and is positively correlated with specific IgE levels [52], [53], [54] and Th2 cytokines [39], [55]; protection is thought to be effected by eosinophils against the incoming larvae using antibody-dependant cellular cytotoxicity [3], [20], [56], [57], [58]. Cook *et al.* [59] recently showed that mice given repeated cercarial infections, before egg deposition, have a Th2 phenotype in the skin with eosinophil infiltration indicating the larvae can also skew towards this response in this animal model. A similar allergic inflammatory skin response was seen in rhesus monkeys given repeated immunisations with radiation-attenuated *S. japonicum* larvae [60]. In this model the skin is the major site of larval attrition during the first 24 h, and larvae were cleared by 3 days [61]. Since buffaloes have a natural and acquired immunity, it is conceivable that the strong inflammatory type-2 response with eosinophil infiltration in the skin region observed here is a mechanism for immunity against penetrating larvae in a similar fashion to that seen in immunised rhesus monkeys. It also reflects what is thought to be the mechanism in human immunity, since human eosinophils have been shown to kill newly-transformed larvae mediated by specific antibody [56].

Unlike in other hosts, IL-10 was not induced in the skin and this suggests the possibility that a local regulatory environment is not induced in buffalo skin, allowing an effective skin response to be induced. Furthermore it is possible that the increased skin thickness of large animal hosts compared to murine skin may delay larval migration and allow more effective immunity to develop at this tissue site. While trapped or killed larvae were not observed in buffalo skin here, dead larvae would most likely be cleared before 5–6 DPI as in immunised rhesus monkeys, and further studies at earlier time points would be required to confirm this.

The present study provides a novel insight into the immune response of water buffaloes, important transmission reservoir hosts for schistosomiasis japonica, and indicates that distinct type-1 and -2 responses occur in distinct tissue regions against migrating larvae, with a strong eosinophil-dominated type-2 response in the skin. Since water buffaloes may be able to generate partial immunity to *S. japonicum*, vaccine formulations replicating the response described in this study may be needed in order to be effective.
